# Chromosomal Evolution in Lower Vertebrates: Sex Chromosomes in Neotropical Fishes

**DOI:** 10.3390/genes8100258

**Published:** 2017-10-05

**Authors:** Marcelo de Bello Cioffi, Cassia Fernanda Yano, Alexandr Sember, Luiz Antônio Carlos Bertollo

**Affiliations:** 1 Departamento de Genética e Evolução, Universidade Federal de São Carlos, São Carlos, SP CEP 13565-905, Brazil; yanocf@ufscar.br (C.F.Y.); bertollo@ufscar.br (L.A.C.B.); 2 Laboratory of Fish Genetics, Institute of Animal Physiology and Genetics, Czech Academy of Sciences, Rumburská 89, Liběchov 277 21, Czech Republic; alexandr.sember@seznam.cz

**Keywords:** alternative evolutionary models, simple and multiple sex chromosomes, independent and common origins, conventional and molecular cytogenetics

## Abstract

Fishes exhibit the greatest diversity of species among vertebrates, offering a number of relevant models for genetic and evolutionary studies. The investigation of sex chromosome differentiation is a very active and striking research area of fish cytogenetics, as fishes represent one of the most vital model groups. Neotropical fish species show an amazing variety of sex chromosome systems, where different stages of differentiation can be found, ranging from homomorphic to highly differentiated sex chromosomes. Here, we draw attention on the impact of recent developments in molecular cytogenetic analyses that helped to elucidate many unknown questions about fish sex chromosome evolution, using excellent characiform models occurring in the Neotropical region, namely the Erythrinidae family and the *Triportheus* genus. While in Erythrinidae distinct XY and/or multiple XY-derived sex chromosome systems have independently evolved at least four different times, representatives of *Triportheus* show an opposite scenario, i.e., highly conserved ZZ/ZW system with a monophyletic origin. In both cases, recent molecular approaches, such as mapping of repetitive DNA classes, comparative genomic hybridization (CGH), and whole chromosome painting (WCP), allowed us to unmask several new features linked to the molecular composition and differentiation processes of sex chromosomes in fishes.

## 1. Introduction

For cytologically distinguishable sex chromosomes to emerge, one of the homologues needs to acquire at least two linked alleles that are advantageous to one sex only and possibly detrimental to the other [1,2]. The next required step is the suppression of recombination between the homologues of the proto-sex chromosomes via chromosomal rearrangements (most commonly inversions or translocations) and/or accumulation of repetitive DNA sequences. In fact, this is a precondition for further gradual genetic and morphological differentiation of the sex pair, through the spreading of the non-recombining region facilitated by additional heterochromatin/repetitive DNA accumulation, genetic degeneration, and, in the long run, size modification of the sex-specific chromosome [1,2,3,4,5,6].

Unlike well-established sex chromosome systems in mammals, snakes, and birds [5,7], fish sex chromosomes often do not progress through the entire set of the aforementioned evolutionary steps [7,8,9,10]. In contrast, many of them are rather of cryptic nature, reflecting perhaps their relatively young evolutionary age [7,11]. Such characteristics often allow fish sex chromosomes to escape from detection under conventional cytogenetic analysis. Consequently, only about 10% of fish species surveyed so far display distinguishable sex chromosomes, with about half of them belonging to the Neotropical region [9,10,12,13]. Such ichthyofauna with approximately 6000 freshwater fish species, represent the world’s richest one [14].

Remarkably, even considering the small number of known cases, several different sex chromosome systems have been described for diverse fish taxa [13,15,16], demonstrating their high evolutionary diversity and plasticity. Overall, at least nine distinct sex chromosome systems have already been recognized among fishes, including the primary ♀XX/♂XY, ♂ZZ/♀ZW and the derived ♀XX/♂X0, ♂ZZ/♀Z0 systems, the standard ♀X_1_X_1_X_2_X_2_/♂X_1_X_2_Y, ♀XX/♂XY_1_Y_2_, ♂ZZ/♀ZW_1_W_2_ multiple ones [12,17,18], along with some unique types such as ♂Z_1_Z_1_Z_2_Z_2_/♀Z_1_Z_2_W_1_W_2_ and ♀X_1_X_1_X_2_X_2_/♂X_1_Y_1_X_2_Y_2_ [16,19,20]. The same extent of variability amounts also for the Neotropical ichthyofauna [17,18,21].

Sex chromosomes often emerged independently and at various times in many fish lineages, following distinct patterns of differentiation even in closely related species [7,10,11,12,22,23,24,25]. In fact, only few exceptions are known from this general view, as in the whole genus *Triportheus* [26,27] and some *Leporinus* [28,29], and *Characidium* species [30,31]. Similarly to what occurs among amphibians and reptiles [7,32], sex chromosome turnover is also a common trait in fishes, which may be achieved by (i) the recruitment of a new sex-determining gene on an autosome, (ii) the transposition of a sex-determining locus to an autosome, or (iii) fusion events among autosomes and cryptically differentiated sex chromosomes, giving rise to the so-called neo-sex chromosome systems [7,11,15,32,33]. Meiotic drive and sex-specific selection pressures are also probably evolutionary forces facilitating such turnover [15,32,34,35,36]. In addition, the high lability of fish sex chromosome systems and, in a broader scale, the sex determination mechanisms themselves, may be also related to the high plasticity of teleost genomes after whole-genome duplications [11] or even to the need for adaptation to varying environmental conditions (e.g., to counterbalance sex ratio distortions after the colonization of a new biotope) [37]. Finally, the stable conditions for biochemical reactions in warm-blooded organisms might favor their conservative sex chromosome constitution, unlike the situation in cold-blooded animals, and thus also in fishes [37].

The above-mentioned features qualify some fish species as excellent models to examine sex chromosome evolution, calling for more in-depth cytogenetic and genomic studies. While the identity, structure, and expression of particular genes are still rather poorly explored in fish sex determination and differentiation [35,37,38,39], relevant advances have been achieved concerning the molecular composition and differentiation process of sex chromosomes employing molecular cytogenetic procedures, such as chromosomal mapping of repetitive DNAs, comparative genomic hybridization (CGH), and whole chromosome painting (WCP) (e.g., [25,40,41,42]).

In this sense, our present review emphasizes two alternative models to investigate the evolution of sex chromosomes among Neotropical fishes, exemplified by species from the Erythrinidae family and the *Triportheus* genus from the Triportheidae family. These fish groups have been our investigation systems for years, for which a considerable amount of conventional and molecular cytogenetic data show differential pathways on the differentiation process of the sex chromosomes.

## 2. The Erythrinidae Family: A Broad Scenario on Fish Sex Chromosomes Evolution

Erythrinidae is a small characiform family, widely distributed in the Neotropical region, with only three genera: *Hoplias* (Gill, 1903), *Hoplerythrinus* (Gill, 1895), and *Erythrinus* (Scopoli, 1977), providing a rare opportunity to gain insights into the evolutionary forces that drive the origin of nascent sex chromosomes, the evolution of the sex pair and speciation processes. In fact, the lack of heteromorphic sex chromosomes, as well as the occurrence of sex chromosome systems with distinct evolutionary stages of differentiation, can be found among different populations of the wolf fish *Hoplias malabaricus* and/or the red wolf fish *Erythrinus erythrinus* [24,43]. Noteworthy, these fishes are also unique in the way that males are always the heterogametic sex—an uncommon trait among fishes (reviewed in [9]).

**Figure 1:**
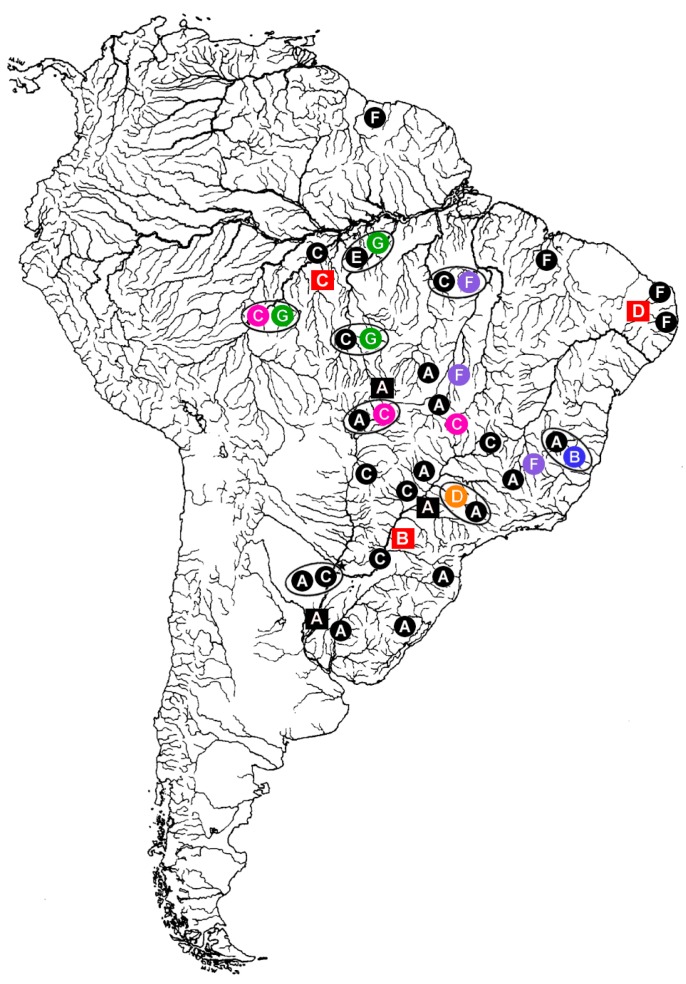
Distribution of *Hoplias malabaricus* (HMA) karyomorphs A–G (circles) and *Erythrinus erythrinus* (ERY) karyomorphs A–D (squares) in the South America. The large open circles indicate some of the sympatric conditions already detected among distinct *H. malabaricus* karyomorphs. The sex chromosome systems until now identified among the distinct karyomoprhs are highlighted in colors, as follows: Black: karyomorphs with homomorphic and/or unidentified sex chromosomes; Blue: the XY sex system of HMA karyomorph B; Pink: the XY sex system of HMA karyomorph C; Orange: the X_1_X_2_Y sex system of karyomorph D; Purple: the XY system of HMA karyomorph F; Green: The XY_1_Y_2_ sex system of karyomorph G; Red: the X_1_X_2_Y sex system of ERY karyomorphs B, C, and D. Note that the occurrence of XY sex systems in HMA karyomorphs C and F are highlighted only for populations where these systems were already investigated. Modified from [24].

**Figure 2:**
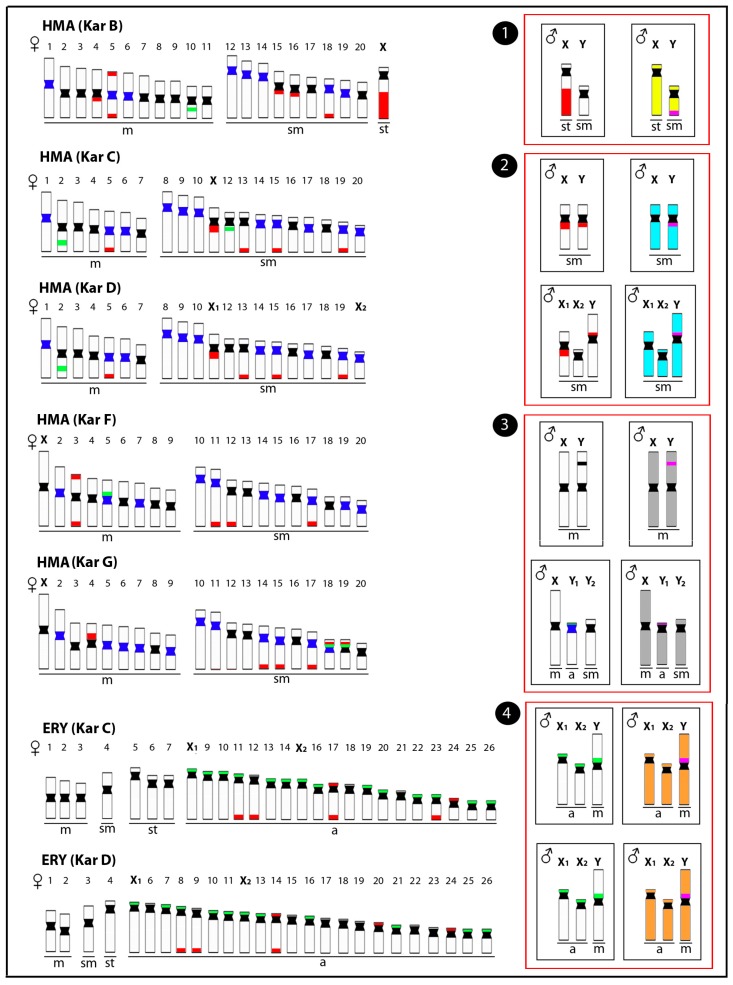
Representative idiograms of Erythrinidae species *Hoplias malabaricus* (HMA) and *Erythrinus erythrinus* (ERY) with differentiated sex chromosomes, highlighting the distribution of different classes of repetitive DNAs and the male-specific regions. The locations of the satellite 5SHindIII-DNA, 18S rDNA, and 5S rDNA sites on the chromosomes are indicated in dark blue, red, and green, respectively. The male-specific regions identified by CGH are highlighted in pink. The sex chromosomes of these species have resulted from at least four independent evolutionary events (red boxes), including (**1**) the XY chromosomes of HMA karyomorph B (yellow), (**2**) the XY chromosomes of HMA karyomorph C and their derived X_1_X_2_Y chromosomes of karyomorph D (light blue), (**3**) the XY chromosomes of HMA karyomorph F and the XY_1_Y_2_ chromosomes of karyomorph G (grey), and (**4**) the X_1_X_2_Y chromosomes of ERY karyomorphs C and D (orange). m = metacentric, sm = submetacentric, st = subtelocentric and a = acrocentric chromosomes. Data from [25,45,50,59,60].

Based on a multitude of chromosomal and molecular data, especially within the last 20 years, it has become increasingly apparent that we are dealing with a group of species instead of a single taxonomic unit, both for *H. malabaricus* and *E. erythrinus* [24,44]. From the cytogenetic standpoint, seven distinct karyotype forms or karyomorphs (A–G) in *H. malabaricus* and four (A–D) in *E. erythrinus* have already been identified, respectively, based on major differences in diploid chromosome numbers (2n), chromosome morphology, and sex chromosomes [44]. Studies employing WCP, CGH, and repetitive DNA distribution patterns have highlighted the differentiation plasticity of the erythrinid sex chromosome systems [25,45,46,47]. More specifically, it was demonstrated that sex chromosomes could emerge via independent pathways, following distinct patterns of differentiation even within the same type of system and among closely related karyomorphs (Figure 1). These features suggest that sex chromosome turnover might play an important role in the speciation process of these fishes (Figure 2).

Within *H*. *malabaricus*, karyomorph B (2n = 42, both sexes) exhibits the most differentiated ♀XX/♂XY sex chromosome system, where the subtelocentric X chromosome is clearly distinguished from the small-sized metacentric Y by a conspicuous heterochromatic block on its long (q) arms [48,49]. Several complementary studies performing mapping of repetitive DNAs, such as 18S rDNA and various microsatellite motifs, reported the unusual enrichment of the X-restricted heterochromatin by such sequences [49,50,51]. These general data, in addition to WCP, indicate that the XY chromosomes of karyomorph B are likely derived from the chromosome pair No. 21 found in karyomorph A [25,52]. Noteworthy, a male-specific region is distally confined to the q arms of Y chromosome, corresponding to a C-positive heterochromatic block occupying the same chromosomal region [47]. Indeed, the location of this region stands out given that a large nucleolar organizer region (NOR) site in the corresponding region of the X chromosome is responsible for the big size difference between both sex chromosomes [48].

Karyomorph C (2n = 40, both sexes), is characterized by morphologically undifferentiated XX/XY sex chromosome system evidenced by a small, but considerable, heterochromatin accumulation on the exclusively proximal region of the X chromosome [53]. CGH results documented the presence of distinct male-specific sequences in this likely nascent sex-determining region [47]). As reported for karyomorph B, the preferential accumulation of repetitive DNAs on the X chromosome is also a particular feature for the nascent XY sex system of this karyomorph. Karyomorph D has 2n = 40 chromosomes in females and 2n = 39 in males, resulting from multiple sex chromosomes of X_1_X_1_X_2_X_2_/X_1_X_2_Y type [54]. Stabilized sex trivalents are found during male meiosis, as well as asynapsis in a presumed sequence divergence region [55], thus pointing to a putative sex-specific region. Rosa et al. [56] reported marked alterations in the location of constitutive heterochromatin and 18S rDNA sites on the Y chromosome, indicating that pericentric inversions probably have also taken place in the early process of sex-specific chromosome differentiation. However, CGH data did not reveal any unique Y-specific region [47]. Therefore, it is possible that yet insufficient sequence divergence within the sex-specific region prevents its detection by CGH, similarly to that recently reported in invertebrates [57].

Karyomorph F (2n = 40, for both sexes) also exhibits a nascent sex chromosome system of XX/XY type [45]. The male-specific content was shown as a prominent interstitial heterochromatic block on the short (p) arms of the large metacentric Y chromosome, also accumulated with several microsatellite motifs and retrotransposons (RTEs), that are absent on the X chromosome [45]. By contrast, karyomorph G (2n = 40 in females/ 41 in males) presents an XX/XY_1_Y_2_ neo-sex chromosomes, where the unusual acrocentric Y_1_ chromosome carries the male-specific region that is also enriched by several types of repetitive DNAs [47].

Similarly to *H. malabaricus*, the species *E. erythrinus* exhibits extensive karyotype diversity among populations, with four karyomorphs (A–D) currently recognized [58]. While karyomorph A is the only one lacking differentiated sex chromosomes, karyomorphs B, C, and D share a multiple X_1_X_1_X_2_X_2_/X_1_X_2_Y sex chromosome system, though they still differ in 2n and karyotype composition. Chromosomal mapping of repetitive DNA by means of FISH (fluorescence in situ hybridization) demonstrated the nature of chromosomal rearrangements and genomic modifications leading to establishment of this X_1_X_2_Y sex system. More specifically, the neo-Y owns its origin to a centric fusion event. In support of this, FISH with telomeric probe highlighted the presence of interstitial telomeric sequences (ITSs) in the centromeric region of the neo-Y chromosome, the only metacentric element in the karyotype [59].

**Figure 3:**
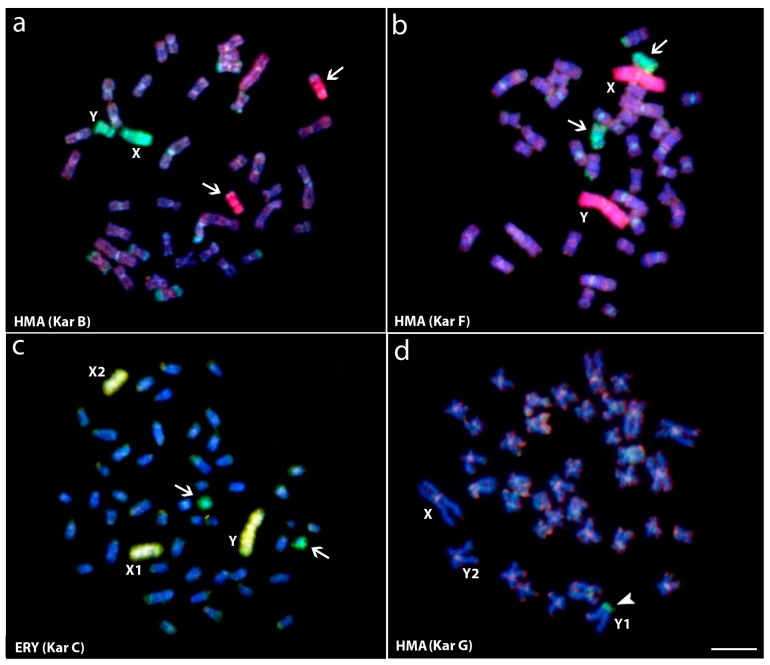
Whole chromosome painting (WCP) and comparative genomic hybridization (CGH) experiments to *Hoplias malabaricus* (HMA) and *Erythrinus erythrinus* (ERY) metaphase chromosomes. (**a**) Male HMA karyomorph B mapped with the X (green) and X_1_ (red) probes from karyomorphs B and D, respectively; (**b**) Male of HMA karyomorph F mapped with the X (green) and the Y (**red**) probes from karyomorphs B and F, respectively; (**c**) Male metaphase of ERY karyomorph D mapped with the X (green) and the Y (yellow) probes, from HMA karyomorph B and ERY karyomorph D, respectively. The arrows indicate the non-homologous chromosomes painted with the sex-specific probes; (**d**) Male of HMA karyomorph G after CGH experiments showing the hybridization pattern of the male-derived probe (green) and female-derived probe (red). Arrowhead points to the male-specific region on the Y_1_ chromosome (**d**). Bar = 10 μm. Data from [25,46].

The repeated occurrence of XY and XY-derived sex chromosome systems in Erythrinidae fishes suggested, at first, a close relationship or even a common origin among them. However, chromosomal painting experiments pointed to at least four independent origins and differentiation processes of the sex chromosomes (Figure 2). A set of whole chromosome probes, including (i) the X chromosome of *H. malabaricus* karyomorph B (HMB-X), (ii) the X_1_ chromosome of *H. malabaricus* karyomorph D (HMD-X1), (iii) the Y chromosome of *H. malabaricus* karyomorph F (HMF-Y,) and (iv) the Y chromosome of *E. erythrinus* karyomorph D (ERY-Y), were used in WCP experiments, providing the pathways of the sex chromosomes differentiation and important hints about interspecific and inter-karyomorph relationships (Figure 3).

The lack of recognizable signals on the *H. malabaricus* karyomorphs B, C, and F sex chromosomes after WCP experiments showed that their XY chromosomes have evolved independently from different autosomal pairs [45,52]. The same approach also identified an autosomal pair from karyomorph A as homeologous to the well-differentiated XY sex chromosomes of karyomorph B and showed that karyomorphs C and D display a close evolutionary proximity. Indeed, in the latter case, the neo-Y chromosome of karyomorph D was proven to arise from a tandem fusion between the nascent Y chromosome and one homologue of the autosomal pair no. 20 present in karyomorph C [53].

Karyomorphs E, F, and G are evidently closely related, thus forming a separate evolutionary clade [43]. The origin of the sex chromosomes in karyomorphs F and G likely resulted from tandem fusion events between acrocentric and submetacentric chromosomes, as found in karyomorph E. In fact, this proposition was strengthened by recent results indicating that in karyomorph F, such rearrangements are found in a homozygous condition, i.e., the large-sized metacentric XY chromosomes, while in karyomorph G, just one homologue per each chromosomal pair had fused and thus giving rise to the unpaired large-sized neo-X chromosome, in addition to the remaining unfused a and sm chromosomes designated as Y_1_ and Y_2_, respectively [46]. Based on these findings, we proposed the parallel evolution of sex chromosome systems of karyomorphs F and G from an E-like karyomorph through different evolutionary scenarios. Mapping of the HMF-Y probe to chromosome complements of other *H. malabaricus* karyomorphs confirmed that sex chromosomes of karyomorph F evolved from different autosome pairs compared to the sex chromosomes of karyomorphs B, C and D [46].

In contrast to *H. malabaricus*, cross-FISH experiments pointed to a common origin of the multiple X_1_X_2_Y sex chromosomes present in *E. erythrinus* karyomorphs, where the centric fusion that gave rise to the neo-Y chromosome probably has occurred before the karyomorphs divergence [61]. However, the same X_1_X_2_Y sex system present in *H. malabaricus* karyomorph D has evolved independently, in which different autosomal pairs were converted to sex chromosomes.

In summary, the representatives of Erythrinidae family serve as a very useful model for studying the emergence and differentiation of the sex chromosomes among fishes. In fact, it highlights a variety of specific scenarios, ranging from emergent to well differentiated sex systems, with a common or independent origins among its clades.

## 3. The *Triportheus* Genus: A Particular Pathway on Fish Sex Chromosomes Evolution 

The *Triportheus* genus (Cope, 1872) comprises 19 extant species popularly known as freshwater sardines [14]. They belong to the monophyletic Triportheidae family, which is also composed of four other genera: *Lignobrycon*, *Engraulisoma*, *Clupeacharax*, and *Agoniates* [62,63]. As to the genus *Triportheus*, its origin dates back to 26.2 ± 6.5 Myr, where *Triportheus auritus* phylogenetically represents the oldest lineage (20.7 ± 6.5 Myr; [63]).

**Figure 4:**
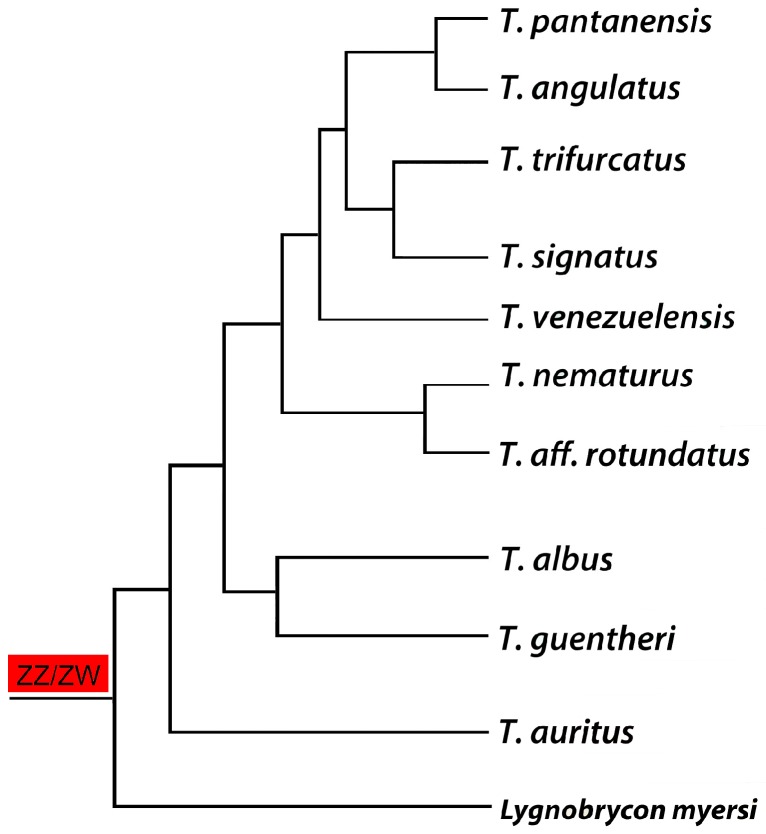
Phylogenetic tree for the *Triportheus* genus (based on the data generated by [63]) highlighting the presence of a ZZ/ZW sex chromosome system in all analyzed species. Note that *Lignobrycon myersi* corresponds to the sister group of all other Triportheidae species and also possesses a similar ZW sex chromosome system, suggesting an early origin of this system in the family.

Similarly to *H. malabaricus* and *E. erythrinus*, this characiform taxon also exhibits a wide distribution throughout South American hydrographic basins [62,64,65]. However, unlike such groups, *Triportheus* exhibits a striking difference of the evolutionary trends dealing with the sex chromosomes. All *Tiportheus* species studied so far share the same sex chromosome system (ZZ/ZW), with evident signs of monophyletic origin (Figure 4). This feature is unique among fishes surveyed to date and it sharply contrasts with the overall picture provided by fish sex chromosome evolution, in which repeated and independent origins, along with frequent turnovers, represent the most common situation documented so far [7,11].

**Figure 5:**
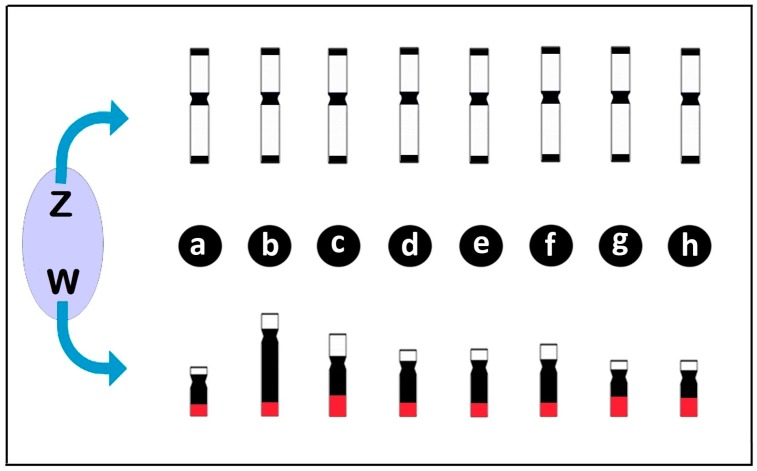
Diagrams illustrating the differentiation of the Z and W chromosomes in *Triportheus* species highlighting the conserved pattern of the Z chromosome in relation to morphology, size and heterochromatin content (in black), in opposite to the divergent patterns of the W chromosome among species. Note that the W chromosome varies in size, morphology, and heterochromatin content, but carries a 18S rDNA cluster on the long (q) arms (in red) in all species. (**a**) *T. albus*; (**b**) *T. auritus*; (**c**) *T. guentheri*; (**d**) *T. nematurus*; (**e**) *T. pantanensis*; (**f**) *Triportheus* aff. *rotundatus*; (**g**) *T. signatus*; and (**h**) *T. trifurcatus*. Data from [42,69,71,76].

To date, 11 out of the 19 *Triportheus* species had been cytogenetically analyzed and all of them share an identical karyotype macrostructure, with 2n = 52 both in males and females [26,27,42,66,67,68,69,70,71,72,73,74,75]. They also share a ZZ/ZW sex chromosome system, with the common metacentric Z chromosome, the largest element of the karyotype. By contrast, the smaller female W chromosome shows interspecific variability in size, morphology, and the content of C-positive heterochromatin [26,67]. The W and Z chromosomes show almost equal size in the most phylogenetically basal species, *T. auritus*, while in the more derived ones, the W chromosome displays a size reduction [42]. Nonetheless, there is no direct correlation between the phylogenetic position and the degree of W degeneration [26,42], nor the most derived taxon in the available recent phylogeny [62] bears the smallest W chromosome. (Figure 5).

Besides the reduction in size, the W chromosome of *Triportheus* has undergone similarly progressive evolutionary dynamics as the Y and W chromosomes of higher vertebrates, with a large accumulation of heterochromatin and repetitive DNA sequences. In this sense, such chromosome is typically almost entirely heterochromatic, while the Z chromosome shows C-positive blocks restricted only to the centromeric and telomeric regions [27,67,68]. In addition, all *Triportheus* species possess characteristically a W-specific 18S rDNA cluster located in the terminal region of the q arms (Wq) [68,72,73,74]. In fact, it has been proposed that the amplification of the rDNA sites on the W chromosome had led to the recombination reduction between the ZW chromosomes [69,75]. Similarly, Reed & Phillips [77] have already suggested a functional importance of NOR on the putative sex chromosome of the Arctic char as it might be able to decrease considerably the recombination rate in the adjacent region (via interference) and thus to facilitate the formation of a possible sex-specific locus there. The location of rDNA sequences within the sex-specific region was already confirmed by FISH and CGH in *T. auritus* [42] and in studies performed in turtles [78,79], while it was alternatively found located adjacent to such region, e.g., in frogs [80]. The maintenance of the 18S rDNA cluster on the W chromosome throughout the evolutionary history of all *Triportheus* species reflects the probable role of these sequences in the evolution of the ZZ/ZW sex chromosome system of this genus [42].

Additionally, the differentiation of the W chromosome in *Triportheus* has been also associated with other repetitive DNA sequences. The W chromosome of *Triportheus albus* also possess another multigene family, the U2 small nuclear RNA (snRNA). Until now, only a few cases are known to occur in fish sex chromosomes (as reviewed in [75]). Microsatellites have also been tightly linked with the W differentiation of *Triportheus* due to their intrinsic ability to rapidly expand in the non-recombinant chromosomal regions [26,74,81,82].

**Figure 6:**
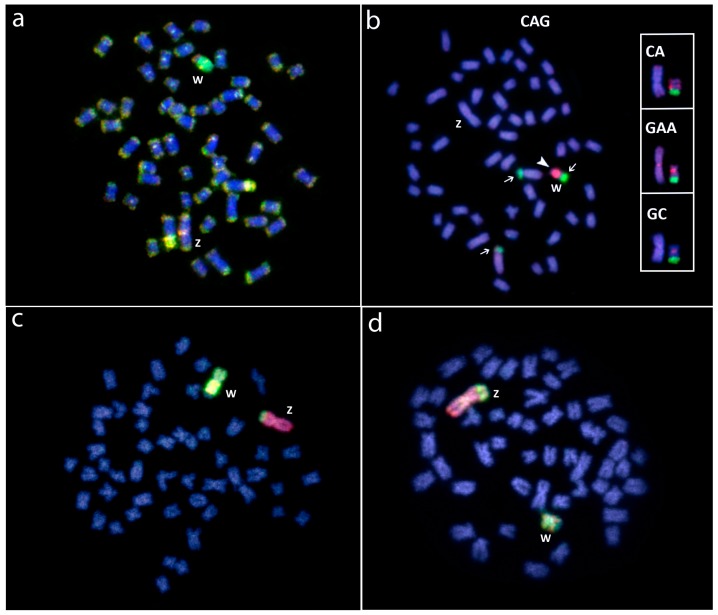
Molecular cytogenetic data in *Triportheus* species. (**a**) Female *T. signatus* showing the chromosome superposition of female (green) and male (red) gDNA probes after CGH experiments. Note the large accumulation of female-specific sequences in the W chromosome; (**b**) Female *T. signatus* chromosomes hybridized with the (CAG)_10_ microsatellite. The hybridization pattern on the Z and W chromosomes using (CA)_15_, (CAG)_10_, (GC)_15_ microsatellite probes are boxed. Note the preferential accumulation of these repeats on the W chromosome; (**c**) Whole chromosome painting in female *T. auritus* and (**d**) *T. albus* (d), using W-chromosome (green) and Z-chromosome (red) probes from *T. auritus*, providing evidence for the common origin of the ZW sex chromosome system in *Triportheus*. Bar = 10 μm. Data from [27,42].

The WCP using both W- and Z-derived probes confirmed the hypothesis about the origin of the ZW sex chromosome system within the *Triportheus* genus. In fact, the Z-probe painted the whole length of the Z chromosome equally in all species, showing its conserved nature in both size and genetic content [42,71] (Figure 6). In turn, the W chromosome of all species was strongly painted using the W-probe from the phylogenetically basal species *T. auritus*, demonstrating that the ZW system in *Triportheus* had a common origin within the genus [42]. Moreover, recent findings showing a morphologically similar ZZ/ZW sex system in *Lignobrycon myersi* (Triportheidae) [83], as well as the occurrence of female heterogamety in species from a sister family (Gasterpelecidae) [84], open new perspectives for investigations of the evolutionary history of the sex chromosomes among these groups. (Figure 4). Thus, the presence of a highly conserved ZZ/ZW system with a monophyletic origin place *Triportheus* as a unique model providing a particular scenario of the evolution of sex chromosomes among the Neotropical fishes.

## 4. Concluding Remarks

In the light of recent cytogenetic progresses, we aimed to illustrate here two contrasting scenarios of sex chromosome evolution in Neotropical fishes: (i) representatives of the Erythrinidae family show high variability and multiple origins of their sex chromosome systems, and (ii) representatives of the genus *Triportheus* highlight a case of monophyletic and stable sex chromosome system, but with different stages of degeneration the sex-specific chromosome. Nonetheless, both models share some common properties, such as repetitive DNA sequences playing a substantial role in the differentiation processes of heteromorphic sex chromosomes. In contrast, the multiple neo-sex chromosomes, arising through the structural rearrangements, do not undergo progressive heterochromatinization [9]—a feature also repeatedly documented in other fish groups [8,20,85,86] that presumably has a large impact on the stabilization of the sex multivalents during meiosis [86,87]. In this sense, it appears to be evident that the highest occurrence of XY-derived multiple sex systems reflects the early stage of differentiation of the XX/XY sex system found in most fishes examined so far. In contrast, the more rapid heterochromatinization and repetitive DNA accumulation observed in the otherwise very frequent ZZ/ZW systems would probably hinder the successful formation of stable ZZ/ZW-derived neo-sex systems. In conclusion, the two alternative models illustrated by the Erythrinidae and *Triportheus* species represent only a fraction of possible evolutionary scenarios within the spectacular fish sex chromosome diversity among lower vertebrates.

As illustrated above, the recruitment of WCP and its cross-species application together with CGH-based experiments represent powerful toolbox for deciphering homologies between distinct sex chromosomes on the interspecific or interpopulational level. However, what accounts for such rapid evolution of sex chromosomes among fishes is yet not fully understood. To obtain a more informed and comprehensive picture, next generation sequencing (NGS) data could be harnessed as a valuable option for studying the genetic basis of sex determination, providing opportunities to identify homologies between sex chromosomes across populations and species in a high throughput manner. Besides, searching for genome-specific satellite DNAs and their subsequent FISH mapping might add another layer of investigation and bring a more complex view about the process underlying fish sex chromosomes evolution.
